# Defining and Phenotyping Gastric Abnormalities in Long-Term Type 1 Diabetes Using a Novel Body Surface Gastric Mapping Device

**DOI:** 10.1016/j.gastha.2023.08.005

**Published:** 2023-08-18

**Authors:** William Xu, Armen A. Gharibans, Stefan Calder, Gabriel Schamberg, Anthony Walters, Jia Jang, Chris Varghese, Daniel Carson, Charlotte Daker, Stephen Waite, Christopher N. Andrews, Tim Cundy, Gregory O’Grady

**Affiliations:** 1 Department of Surgery, The University of Auckland, Auckland, New Zealand; 2 Alimetry Ltd, Auckland, New Zealand; 3 Auckland Bioengineering Institute, The University of Auckland, Auckland, New Zealand; 4 Liggins Institute, University of Auckland, Auckland, New Zealand; 5 Department of Gastroenterology, University of Calgary, Calgary, Canada; 6 Department of Medicine, University of Auckland, Auckland, New Zealand; 7 Department of Gastroenterology, North Shore Hospital, Te Whatu Ora Waitemata, New Zealand

**Keywords:** Gastroparesis, Functional Gastrointestinal Disorders, Dyspepsia, Diabetes Mellitus, Electrogastrography

## Abstract

**Background and Aims:**

Diabetic gastroenteropathy is associated with poor glycemic control and morbidity in people with type 1 diabetes (T1D). There is a lack of noninvasive techniques to assess and monitor gastric abnormalities. We aimed to define phenotypes of gastric myoelectrical abnormalities in people with longstanding T1D with and without symptoms using a novel noninvasive body surface gastric mapping (BSGM) device.

**Methods:**

BSGM was performed on people with T1D of >10 years duration and matched controls, employing Gastric Alimetry (Alimetry, New Zealand), comprising of a high-resolution 64-channel array, validated symptom-logging App, and wearable reader.

**Results:**

Thirty-two people with T1D were recruited (15 with a high symptom burden), and 32 controls. Those with symptoms showed more unstable gastric myoelectrical activity (Gastric Alimetry Rhythm Index 0.39 vs 0.51, *P* = .017; and lower average spatial covariance 0.48 vs 0.51, *P* = .009) compared with controls. Symptomatic patients also had a higher prevalence of peripheral neuropathy (67% vs 6%, *P* = .001), anxiety/depression diagnoses (27% vs 0%, *P* = .001), and higher mean hemoglobin A1C levels (76 vs 56 mmol/mol, *P* < .001). BSGM defined distinct phenotypes in T1D participants including those with markedly unstable gastric rhythms (4/32, 12.5%) and abnormally high gastric frequencies (9/32, 28%). Deviation in gastric frequency was positively correlated with symptoms of bloating, upper gut pain, nausea and vomiting, and fullness (R > 0.35, *P* < .05).

**Conclusion:**

Gastric symptoms in people with longstanding T1D correlate with myoelectrical abnormalities on BSGM evaluation, in addition to glycemic control, psychological comorbidities, and peripheral neuropathy. BSGM using Gastric Alimetry identified a range of myoelectrical phenotypes, presenting targets for diagnosis, monitoring, and therapy.

## Graphical abstract

**Figure:**
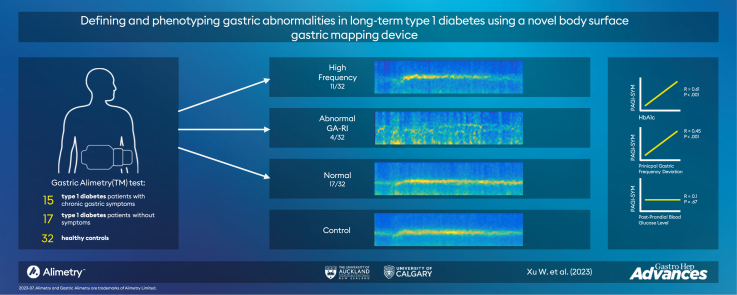

## Introduction

Gastric symptoms are common in type 1 diabetes (T1D), impairing quality of life and compromising nutritional and glycemic control.^1^ Early satiety, pain, nausea and vomiting, and bloating symptoms are often refractory to therapy.^2^^,^^3^ Symptoms are commonly attributed to diabetic gastroparesis, a gastric emptying disorder affecting about 5% of people with T1D.^1^ However, disruptive symptoms without gastric emptying abnormalities or symptoms of nongastric origin frequently occur, leading to the more inclusive term of ‘diabetic gastroenteropathy.’^2^^,^^4^

Autonomic neuropathy, postprandial hyperglycemia, brain-gut axis dysfunction, abnormal gastric emptying, and impaired fundic accommodation are implicated in diabetic gastroenteropathy.^3^ Degradation of interstitial cell of Cajal (ICC) networks and disordered slow wave activity have also been shown in disease, with decreased ICC and rhythm disturbances found in patients with refractory diabetic gastroparesis and chronic nausea and vomiting syndrome.^5^, ^6^, ^7^ Previous studies using electrogastrography (EGG) show frequency and rhythm abnormalities in symptomatic T1D subjects.^8^^,^^9^ However, the use of EGG has not been adopted clinically owing to technical limitations including high sensitivity to ‘noise,’ lack of spatial resolution, and inability to clearly separate disease subgroups.^10^

Body surface gastric mapping (BSGM; high-resolution EGG) is a novel diagnostic technique which overcomes many limitations of EGG.^10^, ^11^, ^12^, ^13^ BSGM employs a dense array of electrodes at the epigastrium, identifying novel biomarkers of slow wave stability and propagation patterns, providing superior symptom correlations with decreased sensitivity to ‘noise.’^11^^,^^12^^,^^14^ These methods may provide insight into the pathophysiology of diabetic gastroenteropathy and offer clinical utility for the noninvasive evaluation of gastric function. The aim of this study was to classify gastric myoelectrical abnormalities and their symptom correlations in people with T1D using noninvasive BSGM.

## Methods

Ethical approval was gained prior to study start (Auckland Health Research Ethical Committee: AHREC:1130; University of Calgary: REB19-1925). All patients provided informed written consent.

People aged ≥18 years with T1D of at least 10 years’ duration, with or without upper gastrointestinal symptoms were recruited in Auckland, New Zealand and Calgary, Canada.

Participants were assessed using the Rome IV criteria for chronic nausea and vomiting syndrome or functional dyspepsia to determine a significant chronic upper gastrointestinal symptom burden^15^ and were stratified into those with and without significant symptoms based on meeting at least one of these criteria. Exclusion criteria included a history of gastroduodenal surgery, active gastrointestinal malignancy, active gastrointestinal infection (including *H pylori*), active neurogenic or endocrine disorders affecting gastric motility (multiple sclerosis, scleroderma, Parkinson’s disease, and hyperthyroidism), current pregnancy, cognitive impairment, cyclical vomiting syndrome, or cannabinoid hyperemesis.

### Patient Matching

Participants with T1D were matched to a database of controls (110 subjects ≥18 years recruited during 2021) in a 1:1 ratio using the nearest neighbor based on age, sex, and body mass index (BMI), with the *matchit* package. Control subjects were excluded if they had active gastrointestinal symptoms, met Rome IV criteria, were taking medications altering gastrointestinal motility, or consumed regular cannabis. Tests with >50% of the duration marked as artifacts were excluded from the analysis, per the Gastric Alimetry Instructions for Use.

### Study Procedure

**Figure 1:**
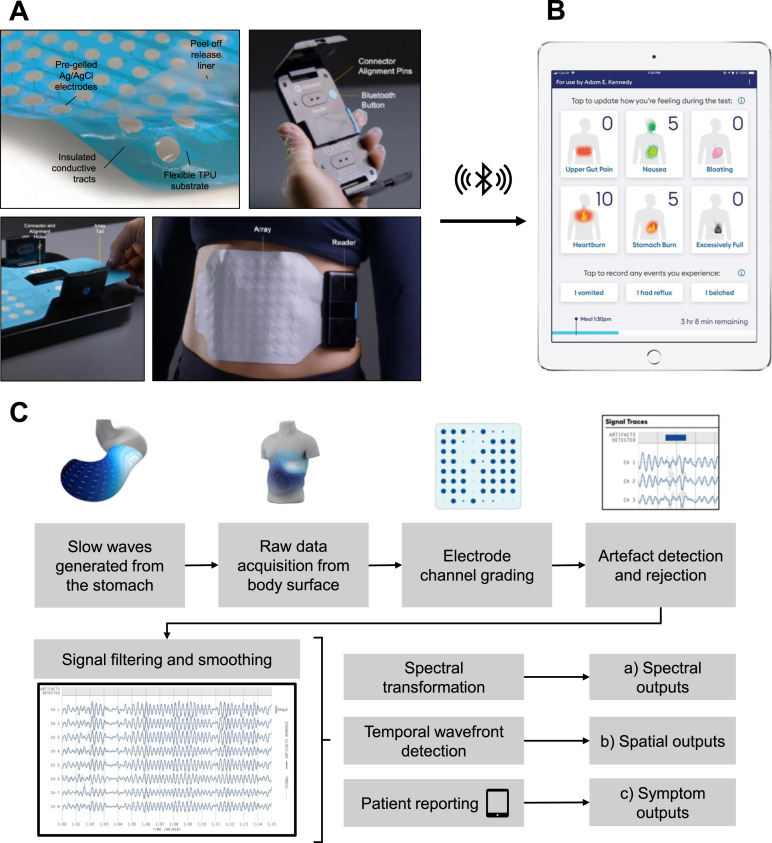
(A) The Gastric Alimetry BSGM system. (B) The Gastric Alimetry App used for setup, data transfer, and symptom data tracking. (C) Signal processing. End outputs include spectral, spatial, and symptom outputs described in Figure A1.

BSGM was performed using Gastric Alimetry (Alimetry, New Zealand), employing a high-resolution 64-channel electrode array, a validated symptom logging App,^16^ and a wearable reader device (Figure 1). The system has been extensively validated to detect gastric myoelectrical activity.^13^^,^^17^^,^^18^

Participants were requested to withhold prokinetic medications, antiemetics, and glucagon-like peptide-1 analogs for at least 24 hours prior to the study, but other medications (insulin, other oral hypoglycemics, antidepressants, anxiolytics, gabapentinoids, and proton pump inhibitors) were not withheld prior to the study. Participants fasted for >6 hours prior to the study, avoiding caffeine and nicotine. After a 30-minute baseline recording, participants consumed a meal including an oat bar and a nutrient drink. Control participants consumed a 68g Clif bar (255 kcal; Clif Bar & Company, CA, USA) and 250 mL of Vanilla Ensure (232 kcal), while participants with diabetes consumed either a 70g Optifast Chocolate bar (234 kcal, Nestle Healthcare Nutrition, NJ, USA) or a 60g Horley’s Cookies and Cream bar (194 kcal; Horleys, Auckland, NZ) and 200 mL of Diasip (208 kcal; Abbott Nutrition, IL, USA). The meal was followed by a 4-hour postprandial recording. Participants sat reclined in a chair with their legs elevated, were asked to limit movement, talking, sleeping, and array touching but were able to read, work on a mobile device, and mobilize for comfort breaks.

### Patient-reported Outcomes

Baseline symptom severity and quality of life was completed with the Patient Assessment of Upper Gastrointestinal Disorders-Symptom Severity Index (PAGI-SYM), Gastroparesis Cardinal Symptom Index (GCSI), and Patient Assessment of Upper Gastrointestinal Disorders-Quality of Life (PAGI-QOL) instruments.^19^^,^^20^ Health psychology assessments were completed using the State Trait Anxiety Inventory Short Form (STAI-SF) and Patient Health Questionnaire-2 (PHQ-2) questionnaires.^21^^,^^22^

Participants rated the severity of upper gut pain, nausea, bloating, heartburn, stomach burn, and excessive fullness every 15 minutes during the test on visual analog scales (0 indicating no symptoms; 10 indicating the worst imaginable symptoms) using the validated Gastric Alimetry App.^16^ Patients rated early satiation immediately after the meal. These data were used to calculate the ‘total symptom burden score.’^16^ Vomiting, belching, and reflux events were tallied.

### Continuous Glucose Monitoring

Continuous glucose monitoring (CGM) was concurrently performed in a subset of people with T1D using Freestyle Libre Pro (Abbott Laboratories, USA) sensors. Sensors were calibrated using capillary blood glucose tests before the study meal, 30 minutes, and 2 hours postmeal (Caresens N, Pharmaco Diabetes, New Zealand).

### Diabetes Complications

The assessment of diabetic complications is detailed in the Supplementary Methods.

### BSGM Analysis

**Figure 2:**
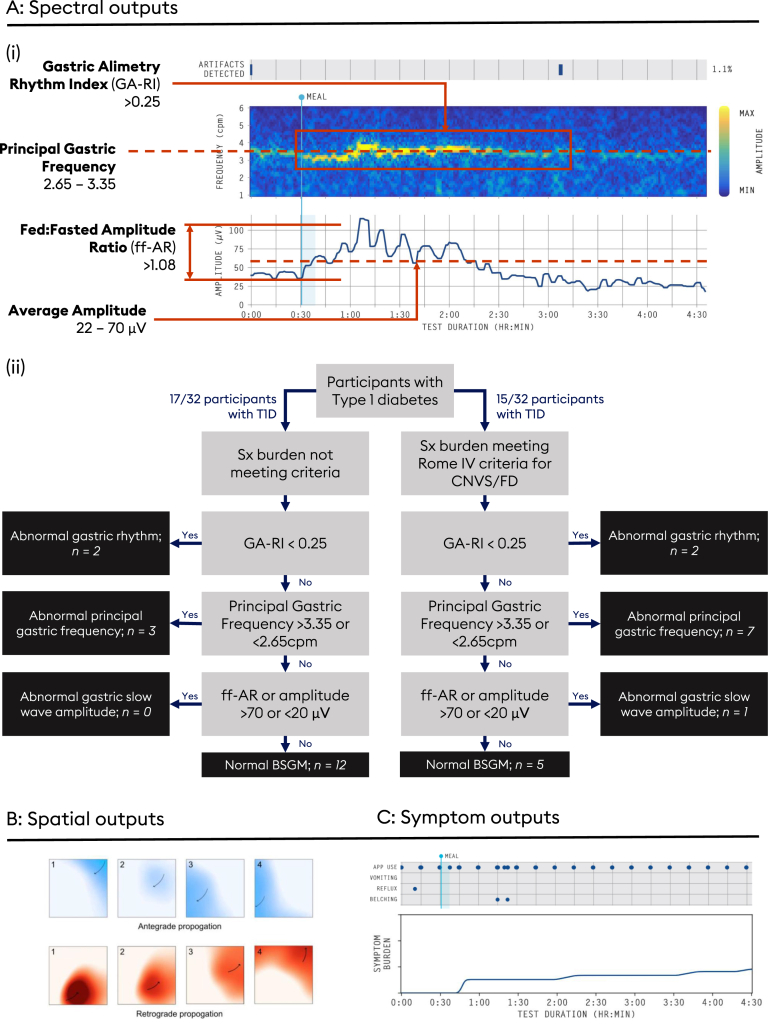
BSGM outputs and classification schematic. The outputs of the BSGM test. (A; i) Spectral outputs include frequency-time spectrograms and amplitude-time curves. Normal gastric conduction produces stable (reference interval Gastric Alimetry Rhythm Index > 0.25) and regular BSGM spectrograms centered on a frequency of 3 cpm (reference interval: 2.65–3.35). A typical meal response results in an amplitude increase after the meal (reference interval Fed:Fasted Amplitude Ratio > 1.08, average amplitude 22–70 μV), before returning toward baseline. (A; ii) Spectrograms of patients can be classified using BSGM normative ranges,^18^^,^^23^ into patients with abnormal gastric rhythm, abnormal gastric frequency, abnormal slow wave amplitude, or normal BSGM. (B) Spatial outputs include phase maps displaying the propagation of gastric slow waves averaged over 15-minute epochs. Frames 1 to 4 denote passage through time. Normal propagation is in the antegrade direction from the gastric fundus toward the gastric antrum and appears as right to left on the body surface.^6^^,^^12^ Retrograde propagation in the opposite direction (B) is associated with pathological states and gastric symptoms.^12^ When no clear antegrade or retrograde pattern was discernible from animations, the corresponding 15-minute epoch was marked as indeterminate. (C) Symptom outputs include a breakdown of symptom-time graphs for each symptom, and the timing of discrete vomiting, reflux, and belching events. Degree of app use can be assessed to ensure quality of symptom logging.

Cohort-level T1D data were compared to matched healthy controls. Individual-level data were compared to reference intervals developed from a recent study of 110 controls.^23^ Four spectral metrics reported by the Gastric Alimetry system were employed (Figure 2A): the Gastric Alimetry Rhythm Index (GA-RI; extent gastric activity is concentrated within a narrow frequency over time relative to the residual spectrum; abnormal <0.25), Principal Gastric Frequency (the sustained frequency associated with the most stable oscillations as defined by GA-RI; normative interval 2.65–3.35 cpm), BMI-adjusted amplitude (normative interval 20–70 μV), and Fed:Fasted Amplitude Ratio (ff-AR, normative interval ≥1.08). Each metric is detailed more in the Supplementary Methods and elsewhere.^18^ Given the bimodal distribution of Principal Gastric Frequency found in the cohort (see Results), Principal Gastric Frequency Deviation (the absolute difference of Principal Gastric Frequency from 3cpm) was calculated to determine abnormality magnitude. Based on the interval ranges, patients were subsequently phenotyped according to their corresponding abnormalities (Figure 2Aii). Assessment of slow wave propagation direction is detailed in Figure 2B.^13^

### Statistical Analysis

Data are reported as the mean ± standard deviation or median and interquartile range unless stated otherwise. Comparisons were made using one-way analysis of variance with further pairwise comparisons via a post hoc Benjamini-Hochberg correction. Fisher’s exact test was used for categorical variables. Continuous independent nonnormal variables were compared using the Mann-Whitney U test. Associations between BSGM metrics, demographic data, CGM data and hemoglobin A1C (HbA1c), and symptoms were assessed using Pearson correlation with Benjamini-Hochberg’s correction to minimize the false discovery rate. A statistical significance threshold of *P* < .05 was used. All analyses were performed in R version 4.0.3 (R Foundation for Statistical Computing, Vienna, Austria).

## Results

BSGM studies were completed in 34 subjects with T1D, of which 2 were excluded for excessive motion artifacts (>50% duration). The remaining 32 subjects were matched by age, sex, and BMI to 32 healthy controls (Table A1). The mean age was 50 years (standard deviation: 14.8) and the majority were women (41/64, 64%). The median BMI was 25.1kg/m^2^ (range: 16.4–38.9) and the median duration of diabetes was 32 years (range: 11–65). All participants with T1D were taking insulin, apart from one symptomatic person with an islet cell transplantation 2 years earlier. History of medication use is detailed in Table A2.

### Symptoms

Of the 32 participants with T1D, 12 met the Rome IV criteria for both chronic nausea and vomiting syndrome and functional dyspepsia, and 3 for functional dyspepsia only, indicative of significant chronic gastric symptoms, while 17 subjects did not meet criteria. Those with gastric symptoms had higher HbA1c levels compared to those without (76 ± 24mmol/mol vs 56±9mmol/mol, *P* = .005). Those with symptoms also had a higher prevalence of anxiety/depression clinical diagnoses (27% symptoms vs 0% no symptoms vs 0% controls; *P* = .010, Table A1), and peripheral neuropathy (67% vs 6%, *P* = .001).

Participants with T1D and gastrointestinal symptoms had higher PAGI-SYM and GCSI scores (all *P* < .001, Figure A1, Table 1). Symptomatic participants had reduced quality of life as measured by the PAGI-QoL score (*P* < .001, Table 1) and demonstrated higher STAI-SF and PHQ-2 scores (all *P* < .05; Table 1).

**Table 1:** BSGM Metrics, Symptom, and Quality of Life Data Between Controls and T1D Patients With and Without Symptoms

Variable	Controls	T1D - no symptoms	T1D - symptoms	Total	*P* value		
					T1D - no sx vs controls	T1D – sx vs controls	T1D - sx -T1D vs no sx
Total N (%)	32 (50)	17 (27)	15 (23)	64	-	-	-
BMI-adjusted amplitude (μV), Median (IQR)	33.3 (27.1–50.0)	35.0 (33.0–40.9)	40.5 (25.7–47.8)	34.9 (27.1–50.0)	.943	.818	.818
Fed:Fasted Amplitude Ratio, Median (IQR)	1.87 (1.47–2.22)	1.80 (1.36–2.26)	1.62 (1.51–2.13)	1.82 (1.42–2.25)	.780	.780	.780
Principal Gastric Frequency (cpm), Median (IQR)	3.09 (2.90–3.24)	3.06 (2.94–3.26)	3.35 (3.08–3.54)	3.12 (2.93–3.30)	.491	.237	.347
Gastric Alimetry Rhythm Index (GA-RI), Median (IQR)	0.51 (0.39–0.75)	0.47 (0.32–0.57)	0.39 (0.26–0.51)	0.47 (0.34–0.61)	.196	.017	.196
Principal Gastric Frequency Deviation, Median (IQR)	0.14 (0.10–0.25)	0.15 (0.06–0.28)	0.41 (0.13–0.54)	0.17 (0.09–0.32)	.523	.048	.106
Average spatial covariance, Median (IQR)	0.51 (0.48–0.55)	0.49 (0.46–0.51)	0.48 (0.46–0.50)	0.50 (0.47–0.52)	.058	.009	.353
Percentage time with retrograde wave propagation (%), Median (IQR)	0.0 (0.0–12.1)	6.7 (0.0–30.6)	7.4 (0.0–19.6)	6.46 (0.00–16.67)	.483	.483	.996
Mean fasting glucose (mmol/L), Median (IQR)	NA	7.43 (6.10–10.07) n = 17	7.87 (7.82–10.65) n = 3	7.82 (6.55–10.07) n = 20	-	-	.368
Mean postmeal glucose (mmol/L), Median (IQR)	NA	9.33 (8.81–12.05) n = 17	8.85 (8.32–10.19) n = 4	9.31 (8.53–12.05) n = 21	-	-	.654
Total symptom burden, Median (IQR)	0.01 (0.00–1.02)	0.34 (0.00–1.43)	11.73 (8.64–16.68)	0.44 (0.00–3.95)	.346	.000	.000
GCSI, Median (IQR)	0.00 (0.00–0.22)	0.11 (0.00–0.44)	2.89 (1.39–3.44)	0.22 (0.00–0.89)	.121	.000	.000
PAGI-SYM score, Median (IQR)	0.10 (0.00–0.31)	0.15 (0.05–0.25)	2.35 (1.18–2.70)	0.22 (0.05–0.60)	.556	.000	.000
PAGI-QoL score, Median (IQR)	0.12 (0.00–0.32)	0.20 (0.03–0.43)	2.31 (1.26–3.43)	0.29 (0.06–0.53)	.260	.000	.000
STAI-SF score, Median (IQR)	13.0 (10.0–15.5)	24.0 (22.0–29.0)	20.0 (16.5–31.0)	19.0 (12.5–24.0)	.000	.007	.955
PHQ-2 score, Median (IQR)	0.0 (0.0–0.5)	0.0 (0.0 to 1.0)	2.0 (1.0 to 3.5)	0.00 (0.00 to 1.00)	.422	.002	.003

*P* values with Benjamini-Hochberg’s corrections for multiple comparisons displayed.

Sx, symptoms; IQR, interquartile range.

### BSGM: Whole Cohort Analysis

BSGM metrics between patient groups are reported in Figure A1 and Table 1, and technical metrics of the test in Table A3. One participant without symptoms had a shortened BSGM test due to threatened hypoglycemia postmeal, while device disconnection and data loss for one hour of the test occurred in another with symptoms. All except for one symptomatic participant finished ≥50% of the study meal, which is sufficient stimulus to generate reliable BSGM metrics (Table A3).^14^ These subjects had adequate meal responses on case review and were kept in the analysis. Overall 80% of patients completed the entire meal (51/64).

#### Rhythm, frequency, and stability

Participants with T1D and symptoms showed marked abnormalities in gastric slow wave stability compared with healthy controls, with reduced GA-RI (0.39 (0.26–0.51) vs 0.51 (0.39–0.75), *P* = .017), higher Principal Gastric Frequency Deviation (0.41cpm (0.13–0.54) vs 0.14cpm (0.10–0.25), *P* = .048), and reduced spatial organization of gastric wave fronts (average ‘spatial covariance’ 0.48 (0.46–0.50) vs 0.51 (0.48–0.55), *P* = .009; Figure A1, Table 1).

In contrast, participants with T1D without symptoms showed minor or no difference compared to healthy controls in GA-RI (0.47 (0.32–0.57) vs 0.51 (0.39–0.75), *P* = .203), Principal Gastric Frequency Deviation (0.15cpm (0.06–0.28) vs 0.14cpm (0.10–0.25) in controls, *P* = .510), and spatial organization of wave fronts (0.49 (0.46–0.51) vs 0.51 (0.48–0.55), *P* = .058; Table 1). Differences in GA-RI, Principal Gastric Frequency, and BMI-adjusted amplitude between T1D participants with and without symptoms were not statistically significant on overall cohort analysis (Figure A1, Table 1).

#### Amplitude

BMI-adjusted amplitude between those with T1D with or without gastric symptoms and healthy controls was similar (median 40.5μV (25.7–47.8) symptoms vs 35.0μV (33.0–40.9) no symptoms vs 33.3μV (27.1–50.0) controls, *P* = .975). Fed:Fasted Amplitude Ratios were comparable (1.62 (1.51–2.13) symptoms vs 1.80 (1.36–2.26) no symptoms vs 1.87 (1.47–2.22) controls; *P* = .927; Table 1.

#### BSGM, symptoms, and glucose levels

Median blood glucose level (BGL) averaged over the 30-minute prestudy period was 7.8 mmol/L (6.6–10.1, n = 20) increasing to 9.3 mmol/L postmeal (8.5–12.0, n = 21; Table 1; Figure A2).

**Figure 3:**
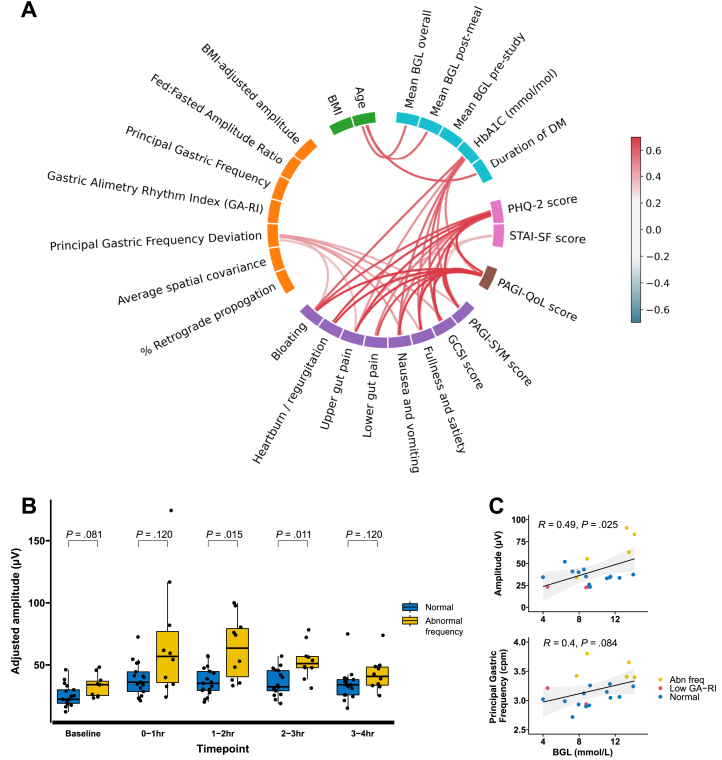
(A) Wheel plot showing correlations. Only statistically significant correlations between categories are shown (after Benjamini-Hochberg correction). (B) Adjusted amplitude between abnormal frequency and normal BSGM subgroups. A T1D participant with abnormal amplitude case is presented in Figure A4. (C) Scatter plot between adjusted amplitude and Principal Gastric Frequency, and blood glucose levels.

Correlation between symptoms, BGLs, and BSGM metrics after Benjamini-Hochberg’s corrections are displayed in Figure 3A. HbA1c, PHQ-2, and STAI-SF scores were positively correlated with GCSI and symptoms such as bloating or nausea and vomiting (R > 0.5, *P* < .05). Principal Gastric Frequency Deviation was positively correlated with GCSI score and symptoms of bloating, upper gut pain, nausea and vomiting, and fullness and satiety (R > 0.35, *P* < .05).

BGLs were related to BMI-adjusted amplitude (R = 0.49, *P* = .025), and Principal Gastric Frequency (R = 0.4, *P* = .084, Figure 3C) on univariate analysis but not after adjustment for multiple comparisons.

**Figure 4:**
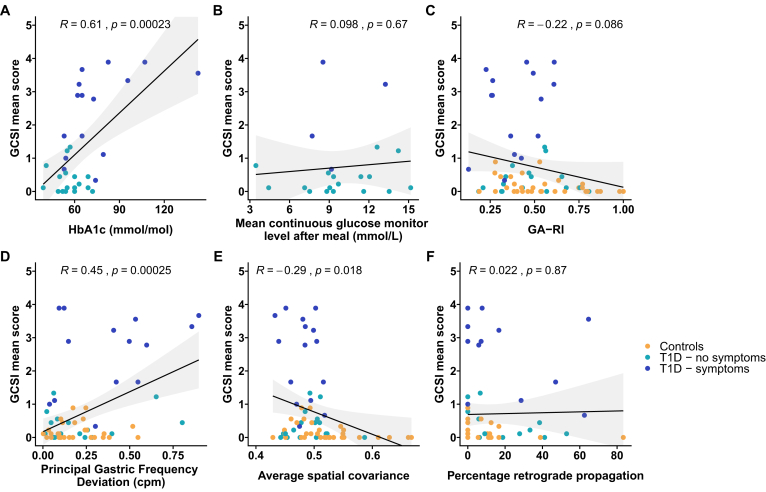
Correlation plots across and those with type 1 diabetes mellitus (T1D) with and without a high symptom burden. Correlations are between GCSI score and (A) HbA1c, (B) mean continuous glucose level after the meal measured by wearable monitors, (C) Gastric Alimetry Rhythm Index (GA-RI), (D) Principal Gastric Frequency, (E) average spatial covariance, and (F) percentage time spent in retrograde propagation. Unadjusted *P* values are shown. CGM data were available for analysis in 20 of 31 diabetic participants (n = 16 minimal symptoms, n = 4 significant upper gastrointestinal [GI] symptoms).

Scatter plots for relationships between BGL, Principal Gastric Frequency Deviation, average ‘spatial covariance’, and symptoms are displayed in Figure 4A–F.

### BSGM: Phenotype Analysis

The classification of spectrograms is presented in Figure 2Aii. BSGM spectral data falling outside reference intervals was more common in people with T1D and symptoms (67% vs 29% without symptoms; *P* = .074).

Using reference intervals, participants with T1D were grouped into phenotypes. Out of all 32 people with T1D, 4 (12.5%) had unstable rhythms, and 10 subjects had abnormal Principal Gastric Frequencies despite normal GA-RI.^18^^,^^23^ One individual (3%) had an isolated high BMI-adjusted amplitude postprandially. A total of 17/32 subjects with T1D (31%) had no abnormality detected on spectral analysis (Figure 2Aii).

**Figure 5:**
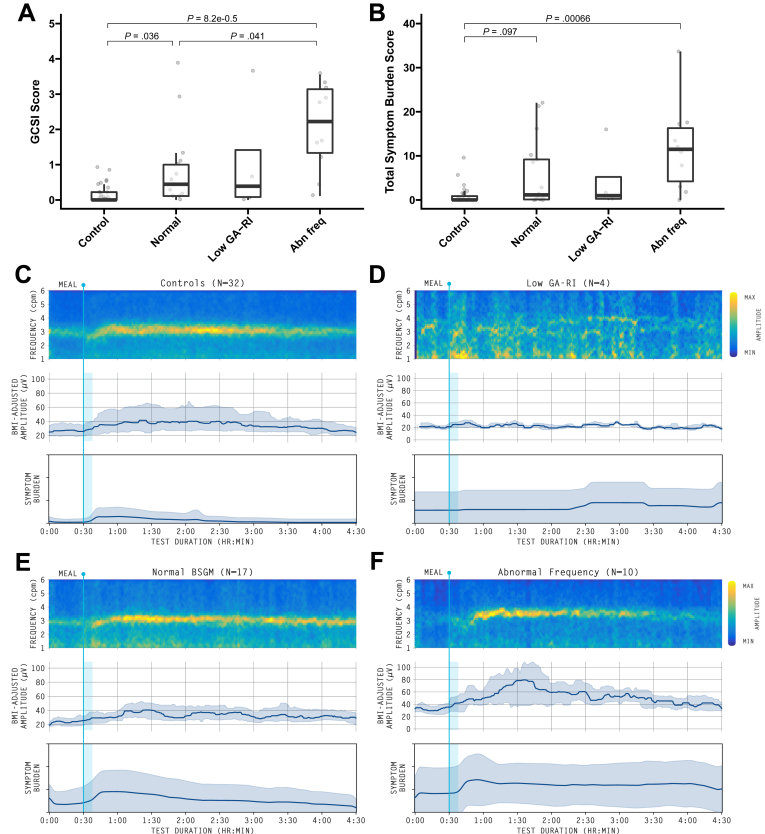
Symptom severity by (A) GCSI score and (B) Total symptom burden,^16^ based on BSGM classification.^18^^,^^23^ (C) Averaged BSGM spectral plots (frequency-amplitude graphs), showing Principal Gastric Frequencies and amplitude, indicating meal responses and rhythms. (C) and (E) Normal spectral plots show clear postmeal power increases, sustained frequency bands, and regular gastric rhythms. (D) Low Gastric Alimetry Rhythm Index (GA-RI) lacked stable activity. (F) Abnormally high or low frequencies (abn freq). Accompanying BMI-adjusted amplitude (median and IQR) and symptom burden plots (mean and SD) shown. SD, standard deviation; IQR, interquartile range.

Out of the 15 symptomatic patients, 2 subjects (13.3%) had unstable rhythms, 6 (40.0%) had high-frequency activity (>3.35 cpm). and one (6.6%) had low-frequency activity (2.5 cpm). One patient (6.6%) had isolated high-amplitude activity. Averaged spectrograms, amplitude plots, and symptom burden during the study across specific phenotypes are displayed in Figure 5A–F.

On spatial pattern analysis, 8 individuals were excluded as >50% of their test duration was classified as spatially indeterminate (3 symptomatic subjects, 2 subjects without symptoms, and 3 controls). There was no overall group-level statistical difference for time spent in retrograde propagation between subjects with and without symptoms or controls (7.4% (0.0–19.6) vs 6.7% (0.0–30.6) vs 0.0% (0.0–12.1), respectively, *P* = .318; Figure A1F, Table A2). Three out of 12 (25%) subjects with T1D and symptoms exceeded the threshold of ≥20% retrograde activity, compared to 5/15 (33.3%) without symptoms and 2/29 (6.9%) controls (*P* = .051).

#### Meal response

People with T1D with Principal Gastric Frequency abnormalities had higher postprandial BMI-adjusted amplitudes than those with normal BSGM (1–2hr: 63.6μV (40.5–79.3) high frequency vs 35.2 (29.5–44.8) normal BSGM, *P* = .015; 2–3hr: 51.1μV (47.8–56.9) vs 32.5 (29.2–45.5), *P* = .011; Figure 3B). Amplitude was more closely correlated with BGL in the abnormal frequency and normal BSGM subgroups compared to the low GA-RI subgroup (Figure A3).

### Sensitivity Analysis

No differences to the main analysis were demonstrated after excluding one subject with symptoms who did not withhold domperidone on the day of the study.

### Safety

No participants experienced a serious adverse event. Mild transient skin redness or rash (n = 10) and itch (n = 5) lasting <48 hours occurred in a subset of participants with spontaneous resolution.

## Discussion

This study applied a new noninvasive medical device (BSGM; Gastric Alimetry) to characterize gastric myoelectrical abnormalities in people with longstanding T1D. Disrupted gastric activity represented by unstable rhythms and abnormal frequencies occurred more regularly in symptomatic patients, with significant correlations to symptoms. Glycemic control, psychological comorbidities, and peripheral neuropathy also correlated with symptoms, reinforcing the multifactorial nature of diabetic gastroenteropathy. Most significantly, Gastric Alimetry enabled phenotyping of T1D gastropathy at the individual patient level based on novel metrics and reference intervals, presenting targets for diagnosis, monitoring, and therapy. Key phenotypes included a group with high Principal Gastric Frequency without gastric rhythm instability (28% of participants) associated with high symptom burden, distinct from a smaller group of participants with highly unstable gastric rhythms (low GA-RI) and lower symptom burdens.

Gastric dysrhythmias are postulated to be key in diabetic gastropathy and are consistently demonstrated in people with T1D suffering symptoms.^8^^,^^12^^,^^24^ However, dysrhythmias are heterogeneous.^25^ Invasive high-resolution serosal mapping studies define specific physiological categories of dysrhythmia, including stable and unstable ectopic pacemaker activity at normal and abnormal frequencies and aberrant spatial conduction pathways, which explain the abnormalities seen noninvasively in the present study.^6^^,^^7^ Legacy EGG studies showed that people with T1D experienced shorter periods in normal gastric rhythms.^8^^,^^26^ However, such results required cautious interpretation as abnormal frequencies measured by legacy EGG can include biologically implausible gastric frequencies (<1.5 or >5cpm), conflating artifacts with stable gastric activity,^27^ such that they could not provide independent measures of frequency and rhythm.^18^ BSGM provides substantially improved capture of gastric myoelectrical activity and has metrics that overcome these limitations. Distinct measures of frequency and rhythm allow more accurate and specific noninvasive assessment of myoelectrical abnormalities in T1D.^18^

The vagus nerve may be important in regulating ICC frequency,^27^ with stable high gastric frequencies demonstrated after vagotomy.^28^^,^^29^ The persistent high frequency phenotype observed in our study is plausibly a manifestation of vagal dysfunction. Vagal nerve pathology in diabetic gastropathy was postulated in the earliest observations by Kassander,^30^ and vagal nerve abnormalities such as reduced myelinated fiber density have been demonstrated in humans, albeit inconsistently.^31^, ^32^, ^33^ Low vagal nerve tone is also associated with impaired gastric accommodation in T1D.^34^ The strong association of peripheral neuropathy and gastric symptoms, in both this study and others, reinforces the presence of a neurogenic mechanism in diabetic gastropathy.^35^ Vagal neuropathy may be one unifying pathophysiological mechanism causing frequency, gastric sensorimotor, and accommodation disturbances in diabetic gastroenteropathy.^2^ Notably, autonomic neuropathy in diabetes often co-occurs with enteric nervous system, ICC, and smooth muscle pathology, such that additional factors could also be contributory in any individual patient.^36^

A smaller subgroup in this study showed a phenotype with weak and unstable gastric myoelectrical activity (low GA-RI) and impaired meal responses, considered to represent gastric neuromuscular abnormalities.^14^ This may reflect underlying depletion and damage to ICC networks,^5^^,^^6^ which are known to contribute to abnormal slow wave dynamics.^6^^,^^7^ Similar myoelectrical disturbances have recently been described in patients with nausea and vomiting syndromes.^14^ Approximately two-thirds of symptomatic T1D patients displayed abnormalities on BSGM, a finding consistent with a recent National Institutes of Health multicenter study which also found abnormalities of gastric myoelectric activity in 66% of gastroparesis and functional dyspepsia patients.^37^ Grover and colleagues identified ICC reductions in 50% of patients with confirmed diabetic gastroparesis on histological analysis, and correlations with gastric emptying data would be of interest in future.^5^ Gastric myoelectric activity differences may also be able to separate patients with gastroparesis and functional dyspepsia into subgroups.^37^^,^^38^ In our study, a number of people with T1D also displayed high amounts of retrograde gastric slow wave activity, something less frequently observed in controls. Sustained retrograde activity has been revealed to be an abnormal feature in gastric disorders and symptom correlations have been demonstrated,^6^^,^^7^^,^^12^^,^^24^ but further work is needed to understand its significance in T1D.

Hyperglycemia is known to influence gastric electrophysiology and motility, potentially inducing dysrhythmia and antral hypomotility. Although blood glucose was associated with Principal Gastric Frequency on univariate analysis in this study, it did not persist after correction for multiple comparisons. Legacy EGG studies have attributed the presence of tachygastria to acute hyperglycemia,^26^^,^^39^ but also have found that tachygastria persists during symptoms despite euglycemia maintenance.^26^ More recent invasive mucosal and high-resolution serosal mapping techniques have revealed that hyperglycemia-induced dysrhythmias are typically transient, occurring during acute changes in blood glucose, and are characterized by spatial and rhythmic disorganization rather than sustained stable high-frequency activity as seen in this study.^40^^,^^41^ Further studies could evaluate blood glucose and its association with gastric abnormalities over longer time periods using emerging wearable devices.^42^

Given that increased amplitude was associated with higher BGLs and those with abnormal frequency had higher gastric amplitudes, one hypothesis is that vagal nerve dysfunction induces rapid gastric emptying,^43^ worsening glycemic control, and raising BGL.^1^ Further studies on this novel finding are needed to determine causality.

Here, several participants with diabetes had normal BSGM testing, with a minority showing normal gastric activity despite the presence of symptoms. Furthermore, on overall cohort analysis, there were no significant differences in BSGM metrics between T1D subjects with and without symptoms, likely reflecting the heterogeneous pathophysiology of gastric symptoms in this group, with a number of patients displaying features of possible gut-brain disorders or visceral hypersensitivity rather than underlying gastric dysfunction.^2^ One person had high gastric amplitude of unknown significance.

Associations between psychological comorbidity and symptoms are not surprising. Complications of diabetes, poor glycemic control, and psychological comorbidities often overlap,^44^ and poor glycemic control frequently coexists with gastric symptoms.^45^ Our findings underscore that psychological comorbidity is common, and may contribute to gastrointestinal sensorimotor dysfunction independently through the gut-brain axis, indicating another area of therapeutic importance.^2^

People with gastric symptoms in diabetes have been previously assessed with transit studies, and classed as gastroparesis, rapid gastric emptying, or having normal gastric emptying.^46^ Gastric emptying remains an important assessment modality in diabetic gastropathy but has come under controversy due to the inconsistent relationship between emptying rate and symptoms, the lack of resolution of symptoms despite emptying improvements, and the variable repeatability over time.^46^, ^47^, ^48^ While this study focused on myoelectrical activity and gastric emptying was not assessed, future studies using both BSGM and gastric emptying simultaneously would be of interest to evaluate how BSGM phenotypes and gastric emptying compare.

Several limitations are acknowledged. Although most participants had BGL monitoring to account for the effects of hyperglycemia, controls and some participants with T1D did not receive CGMs due to device availability. One subject with T1D did not have prokinetic medication withheld, but a sensitivity analysis revealed no significant differences. Although meals given to participants with diabetes were different, the caloric load and volume of the meals remained comparable. Furthermore, reference values for emerging spatial metrics are still being developed, meaning that a consensus-based classification was required. Lastly, the study was limited by study size and further studies with longitudinal BSGM testing are needed. Future work applying BSGM phenotypes to patient treatment pathways will deepen our understanding of the clinical significance of these present findings.

## Conclusion

Gastric symptoms in people with T1D correlate with myoelectrical abnormalities detected with a novel BSGM medical device. Other factors associated with symptoms included glycemic control, psychological comorbidities, and peripheral neuropathy. Most notably, BSGM using Gastric Alimetry was able to identify separate disease phenotypes in T1D characterized by abnormal rhythm stability and abnormal gastric frequencies, representing distinct targets for diagnosis, monitoring, and therapy.

## Supplementary Materials

BSGM in T1D Supplementary Materials
