# Supplementary material for: Defining and Phenotyping Gastric Abnormalities in Long-Term Type 1 Diabetes Using a Novel Body Surface Gastric Mapping Device
Source: Gastro Hep Adv. 2023 Aug 18;2(8):1120–32. doi: 10.1016/j.gastha.2023.08.005 (PMC11307485; doi:10.1016/j.gastha.2023.08.005)
Supplement: BSGM in T1D Supplementary Materials [file mmc1.docx]

**Defining and phenotyping gastric abnormalities in long-term type 1 diabetes using a novel body surface gastric mapping device**

*Phenotyping gastric abnormalities in type 1 diabetes*

**Authors:** William Xu^1^, Armen A. Gharibans^1,2,3^, Stefan Calder^1,2^, Gabriel Schamberg^1,2^, Anthony Walters^4^, Jia Jang^1^, Chris Varghese^1^, Daniel Carson^1^, Charlotte Daker^2^, Stephen Waite^2^, Christopher N Andrews^2,5^, Tim Cundy^6^, Gregory O’Grady^1,2,3^

**Affiliations**

1. Department of Surgery, the University of Auckland, New Zealand
2. Alimetry Ltd, Auckland, New Zealand
3. Auckland Bioengineering Institute, The University of Auckland, New Zealand
4. Liggins Institute, University of Auckland, New Zealand
5. Dept of Gastroenterology, University of Calgary, Canada
6. Department of Medicine, University of Auckland, New Zealand

**Supplementary materials**

**Contents**

[Supplementary methods 1](#_Toc130318960)

[Variables of interest 1](#_Toc130318961)

[Assessment of diabetes complications 2](#_Toc130318962)

[Spatial and spectral data analytics 2](#_Toc130318963)

[Strobe checklist 3](#_Toc130318964)

[Supplementary Tables and Figures 4](#_Toc130318965)

[Table S1: Patient demographics and clinical features of diabetes 4](#_Toc130318966)

[Table S2 : Medication and nicotine use 6](#_Toc130318967)

[Table S3: Test quality 7](#_Toc130318968)

[Table S4: BSGM metrics 8](#_Toc130318969)

[Figure S2: Blood glucose levels (BGL) as measured by continuous glucose monitors during the study. 33](#_Toc130318970)

[Figure S3: Association between Principal Gastric Frequency, amplitude and blood glucose levels 34](#_Toc130318971)

[Figure S4: BSGM of T1D patient with abnormally high amplitude 35](#_Toc130318972)

[Supplementary references 36](#_Toc130318973)

# Supplementary methods

## Variables of interest

Basic information including age, sex and BMI was collected from each participant. Clinical variables included regular medications, duration of diabetes diagnosis, HbA1c within 1-year (mmol/mol), daily insulin use, insulin pump use, and presence of diabetic complications such as neuropathy, retinopathy, nephropathy and cardiovascular disease.

**Study procedure**

## Assessment of diabetes complications

*Retinopathy*

Retinopathy was diagnosed based on ophthalmology clinical reports and classified as either no disease, mild to moderate diabetic retinopathy, or severe disease (severe non-proliferative or pre-proliferative diabetic retinopathy or proliferative retinopathy).

*Nephropathy and neuropathy*

Nephropathy was graded based on estimated glomerular filtration rate (eGFR) thresholds and clinical records (1). A diagnosis of peripheral neuropathy was made based on clinical electronic records as documented by endocrinology or neurology consult notes.

*Cardiovascular and autonomic dysfunction*

Patients had a diagnosis of hypertension and ischemic heart disease recorded from clinical records. Orthostatic hypotension was assessed as a screening tool for sympathetic nervous system dysfunction and was defined as >20mmHg drop in systolic blood pressure or >10mmg drop in diastolic after 5 minutes supine and 1 minute of upright standing (2).

## Spatial and spectral data analytics

***SPECTRAL ANALYSIS***

BSGM spectrograms visualize the bioelectrical slow waves that coordinate gastric activity, as well as amplitude changes which represent meal-responses (**Figure S1**). Four revised BSGM metrics have recently been developed to overcome several pitfalls of traditional EGG metrics (10), providing for accuracy improvements on top of the other advantages of BSGM over traditional EGG, including greater coverage over the stomach area to account anatomical variation of stomach location, a larger number of electrodes, modern bio-amplifiers, and validated signal processing techniques to decrease noise including signals from competing biological sources (3,7).

The revised BSGM metrics include BMI-Adjusted Amplitude, Principal Gastric Frequency, Gastric-Alimetry Stability Index (GA-RI), and Fed:Fasted Amplitude Ratio. Detailed descriptions of metrics are presented in **Table S4**. Normative ranges for these revised BSGM spectral metrics were developed from a cohort of 110 health controls (11).

Patient phenotyping, as described in the methods, was subsequently completed by comparing individual subject-level data with these reference ranges.

***SPATIAL ANALYSIS***

The high-resolution electrode array was used to derive metrics to detect abnormal gastric slow wave activation patterns (12–15). The spatial metrics assessed in this study included ‘average spatial covariance’ and the percentage duration of retrograde wave propagation during the Gastric Alimetry test (3,7,14).

As reference intervals for BSGM spatial metrics are still under development, spatial patterns in 15-minute animation epochs were classified by consensus (four blinded expert assessors) as antegrade, retrograde, or indeterminate (no definite slow wave propagation direction or no consensus) according to the methods of Gharibans et al (7). Only cases with sufficiently high GA-RI (>0.25) and a low percentage of indeterminate periods on consensus classification (<50%) were included in spatial metrics analysis. Example visualizations of phase map animations are displayed in **Figure S1.**

Average spatial covariance was defined by the average absolute value of the covariance between pairs of adjacent electrodes computed over the course of a Gastric Alimetry test.

## Strobe checklist

This study was reported according to the STROBE statement (16).

# Supplementary Tables and Figures

## Table S1: Patient demographics and clinical features of diabetes

| Variables | | Controls | T1D - no symptoms | T1D - symptoms | Total | P value |
| --- | --- | --- | --- | --- | --- | --- |
| Total N (%) | | 32 (50) | 17 (27) | 15 (23) | 64 |  |
| Age | Mean ± SD | 47.8 ± 14.9 | 56.2 ± 14.6 | 47.5 ± 13.5 | 50.0 ± 14.8 | .123 |
| Sex | Female (%) | 20 (62.5) | 11 (64.7) | 10 (66.7) | 41 (64.1) | .960 |
| BMI | Mean ± SD | 25.3 ± 4.1 | 26.2 ± 3.6 | 25.1 ± 6.3 | 25.5 ± 4.5 | .772 |
| Gastroesophageal reflux disease | (%) | 0 (0) | 1 (6) | 0 (0) | 1 (2) | .246 |
| Hypothyroidism | (%) | 0 (0) | 4 (24) | 2 (13) | 6 (9) | .022 |
| Ischemic Heart Disease | (%) | 0 (0) | 2 (12) | 0 (0) | 2 (3) | .058 |
| Hypertension | (%) | 0 (0) | 3 (18) | 0 (0) | 3 (45) | .013 |
| Anxiety/Depression diagnosis | (%) | 0 (0) | 0 (0.0) | 4 (27) | 4 (6) | .001 |
| PTSD | (%) | 1 (3) | 1 (6) | 3 (20) | 5 (8) | .125 |
| Previous non-gastric abdominal surgery | (%) | 12 (39) | 8 (47) | 7 (50) | 27 (44) | .734 |
| Duration of diabetes diagnosis (years) | Mean ± SD |  | 33 ± 15 | 30 ± 15 | 32 ± 15 | .576 |
| HbA1c (mmol/mol) | Mean ± SD |  | 56 ± 9 | 76 ± 24 | 65 ± 20 | .005 |
| Insulin dose (units/day) | Mean ± SD |  | 42 ± 19 | 55 ± 38 | 47 ± 29 | .257 |
| Insulin pump use | (%) |  | 3 (18) | 4 (27) | 7 (22) | .851 |
| Retinopathy - grade | None (%) |  | 7 (41) | 6 (40) | 13 (41) | .986 |
|  | Mild/moderate (%) |  | 7 (41) | 6 (40) | 13 (41) |  |
|  | Severe (%) |  | 3 (18) | 3 (20) | 6 (19) |  |
| Chronic kidney disease - grade | No CKD (%) |  | 17 (100.0) | 10 (66.7) | 27 (84.4) | .152 |
|  | Stage 2-3 (%) |  |  | 4 (27) | 4 (27) |  |
|  | Stage 4-5 (%) |  |  | 1 (7) | 1 (3) |  |
| Peripheral neuropathy | (%) |  | 1 (6) | 10 (67) | 11 (34) | .001 |
| Postural hypotension | (%) |  | 1 (6) | 6 (40) | 7 (22) | .057 |

## Table S2: Medication and nicotine use

One participant with T1D did not withhold their domperidone on the study day. One participant with T1D was on pancreatic enzyme replacement taking Creon.

* Denotes at least once in the last 3 months but not in the last 48 hours prior to the study

| **Variable** | | **Controls** | **T1D - no symptoms** | **T1D - symptoms** | **Total** | **p** |
| --- | --- | --- | --- | --- | --- | --- |
| Total N (%) | | 32 (50) | 17 (27) | 15 (23) | 64 |  |
| Nicotine Use* | (%) | 0 (0) | 0 (0) | 2 (13) | 2 (3) | **0.034** |
| Cannabis use* | (%) | 2 (6) | 0 (0) | 1 (7) | 3 (5) | 0.565 |
| SLGT2 Inhibitor use | (%) | 0 (0) | 2 (12) | 2 (13) | 4 (6) | 0.116 |
| Metformin use | (%) | 0 (0) | 0 (0) | 1 (7) | 1 (2) | 0.190 |
| Prokinetic use | (%) | 0 (0) | 0 (0) | 5 (33) | 5 (8) | **<0.001** |
| Pain neuromodulator use | (%) | 0 (0) | 1 (6) | 5 (33) | 6 (9) | **0.001** |
| Opioid use | (%) | 0 (0) | 0 (0) | 2 (13) | 2 (3) | **0.034** |
| Selective serotonin reuptake inhibitor, benzodiazepine use | (%) | 0 (0) | 1 (6) | 4 (27) | 5 (8) | **0.006** |
| PPI use | (%) | 3 (9) | 4 (24) | 6 (40) | 13 (20) | **0.048** |
| Antiemetic use | (%) | 0 (0) | 0 (0) | 2 (13) | 2 (3) | **0.034** |
| Laxative use | (%) | 0 (0) | 0 (0) | 1 (7) | 1 (2) | 0.190 |

## Table S3: Test quality

| **Variable** | | **Controls** | **T1D - no symptoms** | **T1D - symptoms** | **Total** | **p-value** | | |
| --- | --- | --- | --- | --- | --- | --- | --- | --- |
|  |  |  |  |  |  | **T1D - no symptoms vs Controls** | **T1D – symptoms vs Controls** | **T1D - symptoms-T1D vs no symptoms** |
| Total N (%) | | 32 (50) | 17 (27) | 15 (23) | 64 | - | - | - |
| Impedance (kΩ) | Mean ± SD | 107.9 ± 78.1 | 136.7 ± 66.7 | 186.6 ± 100.1 | 134.0 ± 85.8) | 0.183 | 0.040 | 0.173 |
| Marked artifact (% duration of study) | Mean ± SD | 17.16 (11.75) | 27.81 (19.41) | 29.80 (16.68) | 22.95 (16.13) | 0.000 | 0.000 | 0.506 |
| >50% meal completion | n (%) | 32 (100) | 17 (100) | 14 (93) | 63 (98) | 0.234 | | |

## Table S4: BSGM metrics

*Adapted from Schamberg et al. 2022 ^10^*

| Metric | Description and rationale | Lower | Upper |
| --- | --- | --- | --- |
| BMI-Adjusted Amplitude (μV) | The amplitude/power (μV/dB) associated with dominant frequency in the overall spectrum is confounded by BMI. Gastric Alimetry therefore employs a conservative BMI-adjusted amplitude using a multiplicative regression. | 20 | 70 |
| Principal Gastric Frequency (cpm) | Dominant frequency calculations based on the highest average power across spectra are susceptible to transient bursts of low-frequency signal <2cpm, conflating non-gastric signals with gastric activity.^10^ The ‘principal gastric frequency’ metric instead identifies only the frequency associated with the most stable oscillations, as measured by a distinct new stability metric (GA-RI; see below). The principal gastric frequency therefore detects the intrinsic gastric frequency, as opposed to simply calculating the frequency with the highest average power including all spectral contents whether gastric in origin or otherwise | 2.65 | 3.35 |
| Gastric Alimetry Rhythm Index (GA-RI) | Instability coefficient metrics vary in magnitude based on the dominant frequency (explained in further detail in Schamberg et al. 2022 ^10^). The ‘Gastric Alimetry Rhythm Index’ (GA-RI), provides a measure of rhythmic gastric activity stability, by quantifying the extent to which activity is concentrated within a narrow frequency band over time relative to the residual spectrum. This improves on previous stability metrics in that it has no inherent dependence on the dominant frequency. As a result, the GA-RI enables independent assessment of the frequency and stability of gastric activity. Furthermore, the GA-RI includes a conservative BMI adjustment to account for the effect that signal attenuation has on the perceived relative strength of the gastric activity. | 0.25 | - |
| Fed:Fasted Amplitude Ratio | Amplitude increase following a meal stimulus is a characteristic of healthy gastric function. However, timing of the meal response varies. The Fed:Fasted Amplitude Ratio, instead quantifying the observed meal response by taking a ratio of the overall postprandial amplitude averaged over 4 hours to the preprandial amplitude, takes the ratio between the maximum amplitude in *any* *single 1-hour* across a 4-hour postprandial period to the amplitude in the preprandial period. Given that the goal of amplitude/power ratio metrics is to identify an increase in amplitude/power following meal consumption, it is important to have a metric that can quantify this increase across a cohort of subjects with significant natural variation in the timing of the meal response. | 1.08 | - |

**Figure S1: Symptoms and metrics across controls, and those with type 1 diabetes mellitus (T1D) with and without a high symptom burden.**

**
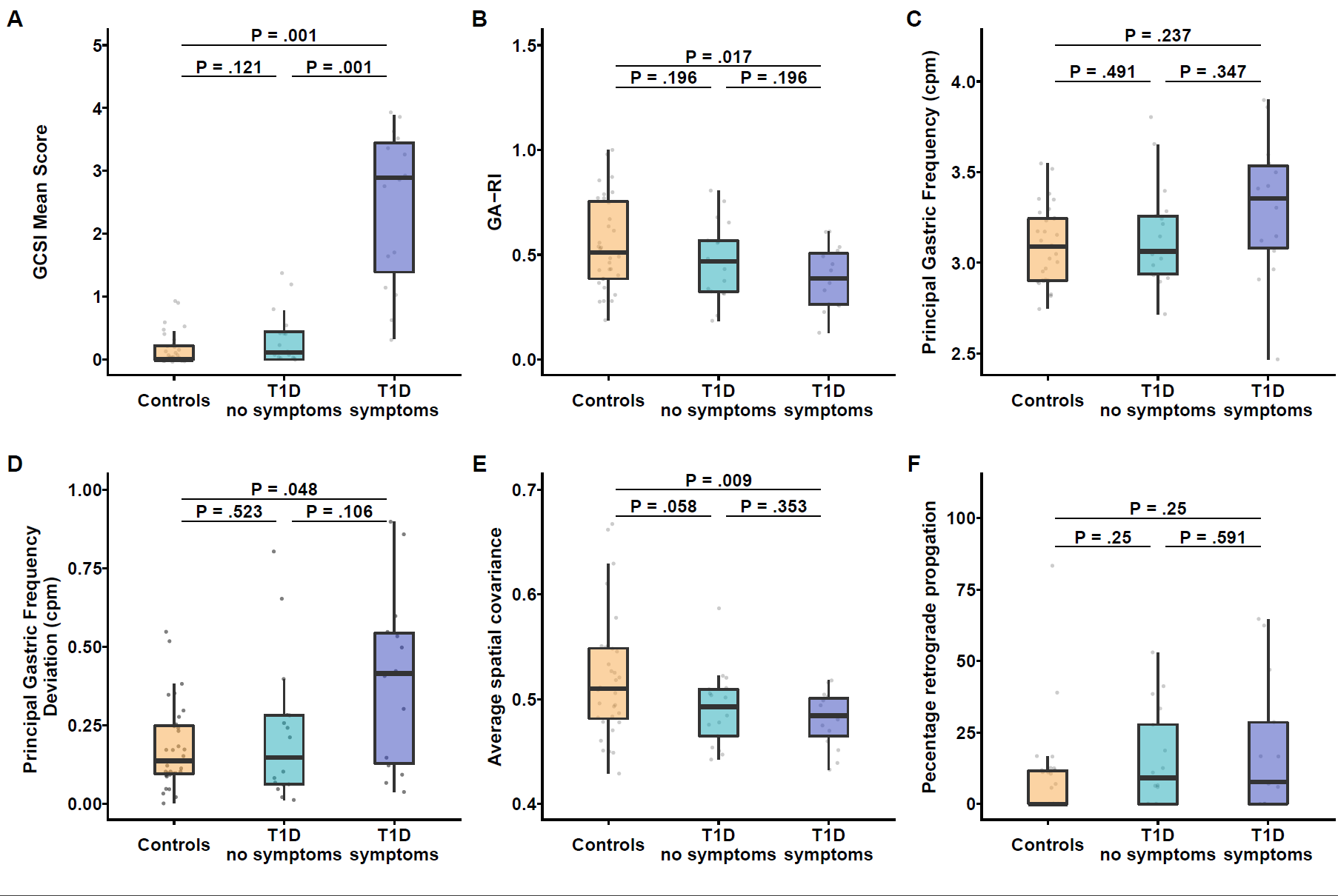
**

## Figure S2: Blood glucose levels (BGL) as measured by continuous glucose monitors during the study.

**
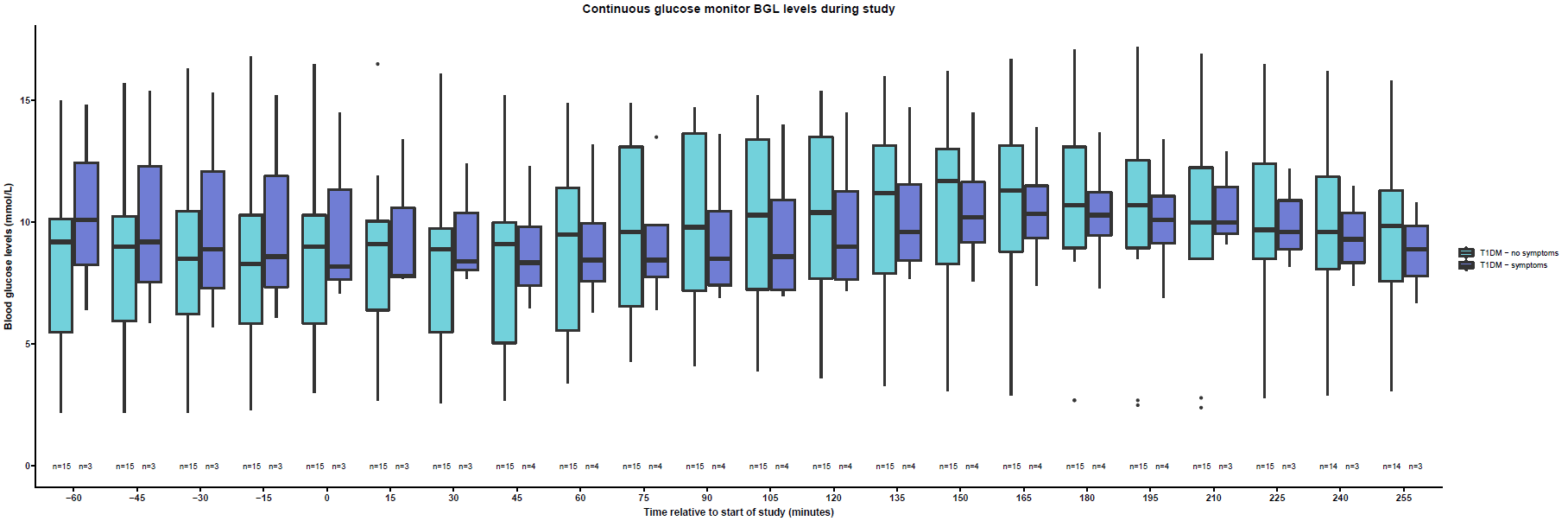
**

## Figure S3: Association between Principal Gastric Frequency, amplitude and blood glucose levels

A) Within-individual pairwise Pearson R correlation coefficients for blood glucose versus amplitude averaged across phenotypes. Averaged across cohorts and compared across groups. Example plots of B) good within individual correlation between amplitude and BGL (r=0.736, p<0.001), C) poor correlation with a delayed BGL peak (r = 0.025, p=0.679), D) poor correlation with a high baseline BGL. BGL, blood glucose levels (r = -0.07, p =0.228).

**
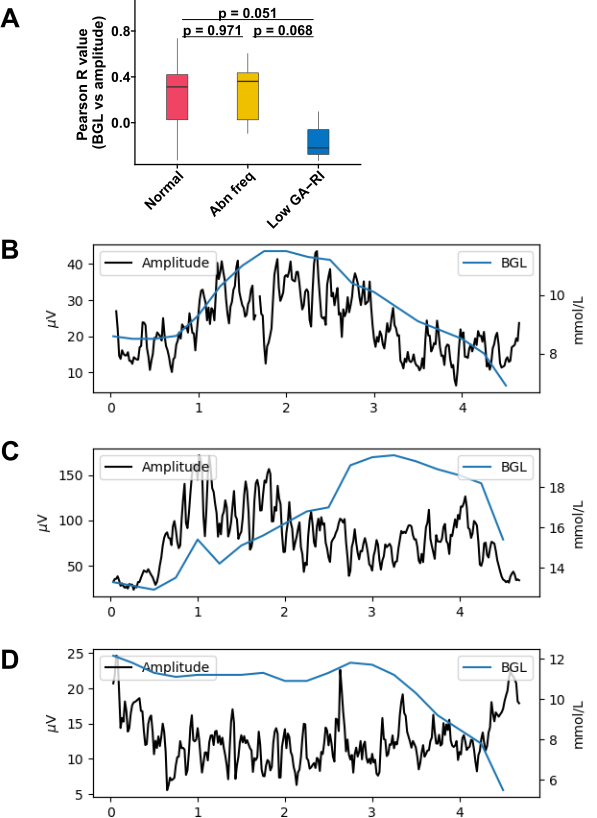
**

## Figure S4: BSGM of T1D patient with abnormally high amplitude

Dark blue blocks within the spectrogram denote areas of high artifact. Note the adjusted amplitude scale relative to Figure 5.

**
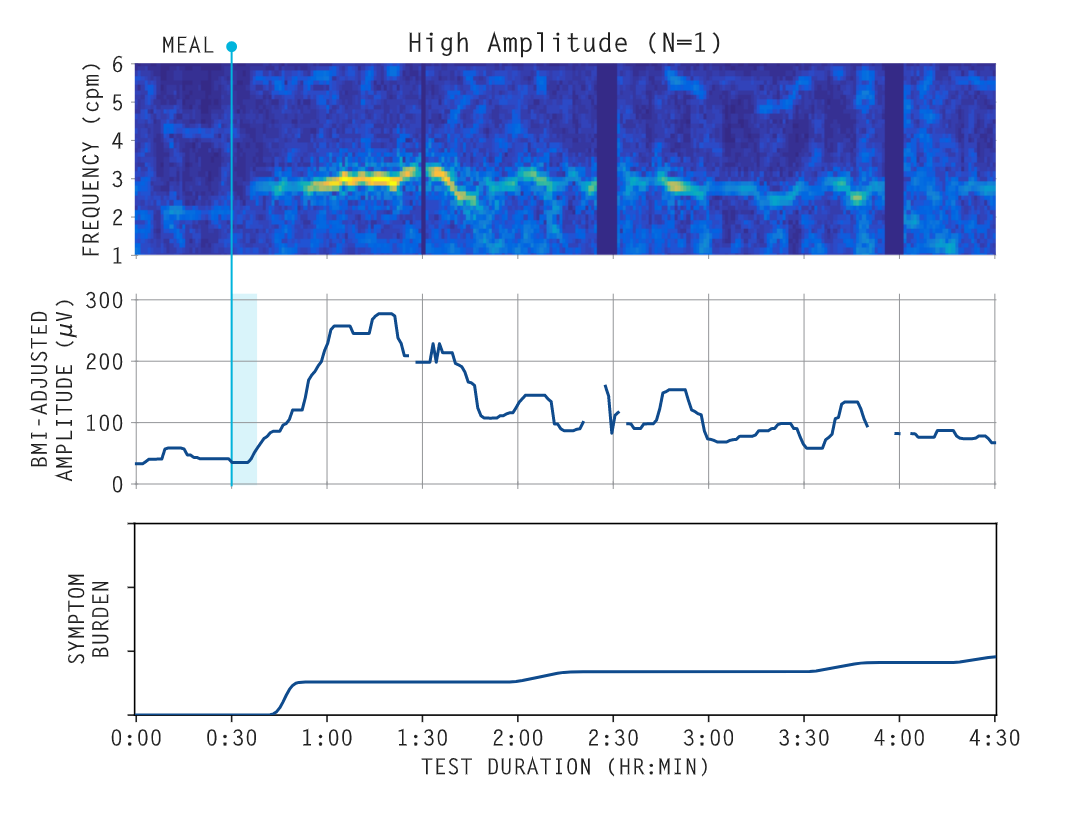
**

# Supplementary references

1. Stevens PE, Levin A, Kidney Disease: Improving Global Outcomes Chronic Kidney Disease Guideline Development Work Group Members. KDIGO 2012 clinical practice guideline for the evaluation and management of chronic kidney disease. Kidney Int [Internet]. 2013; Available from: https://jhu.pure.elsevier.com/en/publications/kidney-disease-improving-global-outcomes-kdigo-ckd-work-group-kdi-4
2. Ricci Fabrizio, De Caterina Raffaele, Fedorowski Artur. Orthostatic Hypotension. J Am Coll Cardiol. 2015 Aug 18;66(7):848–60.
3. Gharibans AA, Calder S, Varghese C, Waite S, Schamberg G, Daker C, et al. Gastric dysfunction in patients with chronic nausea and vomiting syndromes defined by a novel non-invasive gastric mapping device. medRxiv. 2022 Feb 8;2022.02.07.22270514.
4. Gharibans, A. A. Coleman, T. Mousa, H. Kunkel, D. High-density multichannel electrode array improves the accuracy of cutaneous electrogastrography across subjects with wide-ranging BMI: 1235. Am J Gastroenterol [Internet]. 2018; Available from: https://journals.lww.com/ajg/fulltext/2018/10001/high_density_multichannel_electrode_array_improves.1235.aspx
5. Calder S, O’Grady G, Cheng LK, Du P. A Simulated Anatomically Accurate Investigation Into the Effects of Biodiversity on Electrogastrography. IEEE Trans Biomed Eng. 2020 Mar;67(3):868–75.
6. Angeli TR, Du P, Paskaranandavadivel N, Janssen PWM, Beyder A, Lentle RG, et al. The bioelectrical basis and validity of gastrointestinal extracellular slow wave recordings. J Physiol. 2013 Sep 15;591(18):4567–79.
7. Gharibans A, Hayes T, Carson D, Calder S, Varghese C, Du P, et al. A novel scalable electrode array and system for non-invasively assessing gastric function using flexible electronics. Neurogastroenterology & Motility. 2022 Jun 14;e14418.
8. Sebaratnam G, Karulkar N, Calder S, Woodhead JST, Keane C, Carson DA, et al. Standardized system and App for continuous patient symptom logging in gastroduodenal disorders: Design, implementation, and validation. Neurogastroenterol Motil. 2022 Feb 13;e14331.
9. Gharibans AA, Smarr BL, Kunkel DC, Kriegsfeld LJ, Mousa HM, Coleman TP. Artifact Rejection Methodology Enables Continuous, Noninvasive Measurement of Gastric Myoelectric Activity in Ambulatory Subjects. Sci Rep. 2018 Mar 22;8(1):5019.
10. Schamberg G, Varghese C, Calder S, Waite S, Erickson JC, O’Grady G, et al. Revised spectral metrics for body surface measurements of gastric electrophysiology [Internet]. bioRxiv. 2022. Available from: http://dx.doi.org/10.1101/2022.07.05.22277284
11. Varghese C, Schamberg G, Calder S, Waite S, Carson DA, Foong D, et al. Normative values for body surface gastric mapping evaluations of gastric motility using Gastric Alimetry: spectral analysis. medRxiv. 2022 Jul 26;2022.07.25.22278036.
12. O’Grady G, Angeli TR, Du P, Lahr C, Lammers WJEP, Windsor JA, et al. Abnormal initiation and conduction of slow-wave activity in gastroparesis, defined by high-resolution electrical mapping. Gastroenterology. 2012 Sep;143(3):589–98.e3.
13. Angeli TR, Cheng LK, Du P, Wang THH, Bernard CE, Vannucchi MG, et al. Loss of Interstitial Cells of Cajal and Patterns of Gastric Dysrhythmia in Patients With Chronic Unexplained Nausea and Vomiting. Gastroenterology. 2015 Jul;149(1):56–66.e5.
14. Gharibans AA, Coleman TP, Mousa H, Kunkel DC. Spatial Patterns From High-Resolution Electrogastrography Correlate With Severity of Symptoms in Patients With Functional Dyspepsia and Gastroparesis. Clin Gastroenterol Hepatol. 2019 Dec;17(13):2668–77.
15. Somarajan S, Muszynski ND, Olson JD, Comstock A, Russell AC, Walker LS, et al. The effect of chronic nausea on gastric slow wave spatiotemporal dynamics in children. Neurogastroenterol Motil. 2021 May;33(5):e14035.
16. von Elm E, Altman DG, Egger M, Pocock SJ, Gøtzsche PC, Vandenbroucke JP, et al. The Strengthening the Reporting of Observational Studies in Epidemiology (STROBE) statement: guidelines for reporting observational studies. Ann Intern Med. 2007 Oct 16;147(8):573–7.
